# Miscellaneous

**Published:** 1890-09

**Authors:** 


### MISCELLANEOUS.

### TEA AND COFFEE.

Tea is a nerve stimulant, pure and simple, acting like alcohol in this respect, without any value that the latter may possess as a retarder of waste. It has a specific influence upon those nerve centres that supply will power, exalting their sensibility beyond normal activity, and may even produce hysterical symptoms if carried far enough. Its active principle, theine, is an exceedingly powerful drug, chiefly employed by nerve specialists as a pain destroyer, possessing the singular quality of working toward the surface. That is to say, when a dose is administered hypodermically for sciatica, for example, the narcotic influence proceeds outward from the point of injection, instead of inward towards the centres, as does that of morphia, atropia, etc. Tea is totally devoid of nutritive value, and the habit of drinking it to excess, which so many American women indulge in, particularly in the country, is to be deplored as a cause of our American nervousness.

Coffee, on the contrary, is a nerve food. Like other concentrated foods of its class, it operates as a stimulant also, but upon a different set of nerves from tea. Taken strong in the morning, it often produces dizziness and that peculiar visual symptoms of over-stimulus named *muscæ volitantes*—dancing flies. But this is an improper way to take it, and rightly used it is, perhaps, the most valuable liquid addition to the morning meal. It should be made as strong as possible at first in a drip-bag, and a teaspoonful or two of the liquid added slowly to a large cupful of equal parts of hot milk and cream.

### PRESERVING THE HEALTH.

1. Rise early and never sit up late.
2. Wash the whole body every morning by means of a large sponge, and rub it dry with a rough towel.
3. Drink water.
4. Avoid spirits and fermented liquors of every kind.
5. Keep the head cool, and sleep in an airy apartment.
6. Eat no more than enough and let the food be plain.
7. Let your supper be light.

### GIRLS AND WOMEN.

A New York paper recently offered a prize for the best brief answer to the old yet ever new question: "What Shall We Do with Our Girls?" Madame Albani-Gye was judge, and awarded the prize to the writer of a short essay, which proved to be from Ella Wheeler Wilcox. This is the essential part: "The foundation of society rests on its homes. The success of our homes rests on the wives. Therefore, first of all, teach our girls how to be successful wives. Begin in their infancy to develop their characters. Teach them that jealousy is an immorality and gossip a vice. Train them to keep the smallest promise as sacredly as an oath, and to speak of people only as they would speak to them. Teach them to look for the best quality in every one they meet, and to notice other people's faults only to avoid them. Train them to do small things well and to delight in helping others, and instil constantly into their minds the necessity for sacrifice for others' pleasure as a means of soul development. Once given a firm foundation of character like this, which the poorest as well as the richest parents can give to their girls, and no matter what necessity arises they will be able to rise above it."

### AN ENGLISH LEPER AT MOLOKAI.

The London correspondent of the *Birmingham Post* writes ;—I have seen a letter recently received from "Sister Rose Gertrude" concerning her visit to Molokai. After briefly describing the beauties of the island, she refers to the village where Father Damien died. "Here," she says, "we visited a young Englishman, a leper, who was even then entering on his agony. This was one of the most heartrending sights of the island. There, in a little white draped bed by the open lattice, fanned by the southern breeze laden with the fragrance of the jasmine and honeysuckle, lay the sick man, the skin drawn tightly over his bones, one side of his face entirely eaten away by cancer, his eyes bleared and sightless, his hands deformed, his breath coming in quick, short gasps." Sister Rose Gertrude says that the lepers appear to enjoy life in their own way. She met several on horseback, and was assured that in the settlement of 1203 lepers there were 800 horses provided by the government for those who cannot walk. There are three churches in one of the little villages—a Roman Catholic, Calvinist and Moravian.

### HOW TO WASH WINDOWS.

Two servants employed in adjoining houses were talking recently about their methods of cleaning windows. The one whose windows always looked the brightest said she selected a dull day for the work, or a day when the sun was not shining on them, because when the sun shines it causes them to be dry-streaked, no matter how much one rubs. The painter's brush is the best article for this purpose ; then wash all the wood-work before the glass is touched. To cleanse the glass simply use warm water diluted with ammonia ; don't use soap. A small stick will get the dust out of the corners, then wipe dry with a piece of cloth—do not use linen, as the lint sticks to the glass. The best way to polish is with tissue paper or newspaper. To clean windows in this way takes much less time than when soap is used.

### BOSTON BAKED BEANS.

Our Boston friends could hardly exist without their "baked beans and brown bread." We acknowledge their goodness. We know just how it is done. The beans are the little white ones, well cooked without boiling ; that is, simmered slowly till soft, then a little pork, a little salt, a little soda, and a little molasses, and a little water added, and all slowly baked for a long time and allowed to cool in the oven over night, brought to the breakfast table in a very acceptable hot condition. We do not wonder Bostonians like their baked beans and brown bread, too ; for it is, of its kind, hard to beat. It cannot be better than when made after this rule—the genuine old New England style. To one quart of yellow corn meal and one of coarse rye meal add one teaspoonful of salt, one cup of molasses. In one cup of cold water dissolve one heaping teaspoonful of carbonate of soda. Mix this with the other ingredients and also stir in enough cold water to make all into a soft batter. Pour into a well greased pan, steam for three hours or more, then dry off ten minutes in a moderate oven. It is but a few minutes work to make it—to steam it is by far the best way—far superior to baking, and far better than when made with milk.

### THAT WATERLOO BALLROOM.

The approaching seventy-fifth anniversary of the battle of Waterloo will once more revive the interest awakened two years ago by Sir William Fraser in the upper story of the now deserted brewery in the Rue de la Blanchisserie, Brussels, which was beyond a doubt the scene of the Duchess of Richmond's historic "revelry by night." The room is very large, but the rough beams supported by a row of six wooden pillars in the centre can be easily touched by the hand. The rubbish has been cleared away and one can clearly see the traces of the temporary passage by which the Duchess connected her improvised ballroom with her drawing-room in the house, now occupied by the Sœurs Hospitalieres in the Rue des Cendres. M. Vanginderachter, who succeeded Sismon, the coachbuilder, at No. 40, is dead, and next month the building is to be brought to the hammer, the upset price being 192,000f. His widow, a comely Flemish matron, such as Jacques Jordaens would have loved to paint, is inconsolable, for ever since Sir William Fraser's discovery she had been honored with many visitors and has started an autograph album. She fondly hopes the ballroom will find a purchaser among the Duchess of Richmond's compatriots.

### THE ART OF BREAD MAKING.

To make good bread requires but a trifling amount of kneading, and the process of making and baking, as a whole, is not laborious, but rather a pleasant occupation if properly conducted. But four ingredients are required—flour, yeast, salt and water, or milk and water, clear milk or buttermilk. With any one of these good bread can be made. An imperative condition is that the yeast shall be pure and sweet.

More dough is spoiled in baking than in any other way. The temperature of the oven should be from three hundred and fifty to four hundred degrees Farenheit. As thermometers are not usually available, a sufficient test will be that the oven shall throw out a sudden current of hot air when the door is opened ; or that if a few drops of water be sprinkled on the bottom of the oven they will hop and hiss, or that a pinch of dry flour put on the bottom of the oven will become brown, without burning, in two minutes.

A loaf of bread rightly made and baked will be rounded or puffed up in the centre of the upper crust, if the top of the loaf is flat the success is not perfect. When taken from the pan the loaf should be laid where it can cool in the air where there are no odors to be absorbed. The bread should not be wrapped in a cloth and put away, but placed in a bread box which has apertures to admit air. A perfect loaf will remain good for a week or ten days.

### FRIENDS AFTER A FIGHT.

A fine Newfoundland dog and a mastiff had a fight over a bone, or some other trifling matter. They were fighting on a bridge, and being blind with rage, as is often the case, over they went into the water.

The banks were so high that they were forced to swim some distance before they came to a landing-place. It was very easy for the Newfoundland dog ; he was as much at home in the water as a seal. But not so with poor Bruce. He struggled and tried his best to swim, but made little headway.

Old Bravo, the Newfoundland, had reached the land, and turned to look at his old enemy. He saw plainly that his strength was failing and that he was likely to drown. So what should he do but plunge in, seize him gently by the collar and, keeping his nose above water, tow him safely into port.

It was curious to see the dogs look at each other as soon as they shook their wet coats. Their glances said plainly as words : " We will never quarrel any more."

### SALT FOR MOTHS.

Salt is the best exterminator for moths. The nuns in one of the hospital convents have tried everything else without success, and their experience is valuable, as they have so much clothing of the sick who go there, and strangers, when dying there, often leave quantities of clothing, etc. They had a room full of feathers, which were sent there for pillow-making, and they were in despair as they could not exterminate the moths, until they were advised to try common salt. They sprinkled it around, and, in a week or ten days, they were altogether rid of the moths. They are never troubled now. In heavy velvet carpets, sweeping them with salt cleans and keeps them from moths, as particles of salt remain in the carpet and corners.—*Ex.*

IF a person swallows any poison whatever or has fallen into convulsions from having overloaded his stomach, an instantaneous remedy is a heaping teaspoonful of common salt and as much ground mustard stirred rapidly in a teacup of water, warm or cold. It is scarcely down before it begins to come up, bringing with it the remaining contents of the stomach ; and lest there be any remnant of poison, however, let the white of an egg or a teacup of strong coffee be swallowed as soon as the stomach is quiet ; because these very common articles nullify a large number of virulent poisons.

### CHARACTER IN THE FAMILY CIRCLE.

Home life is the sure test of character. Let a husband be cross and surly and the wife grows cold and unamiable. The children grow up saucy and savage as young bears. The father becomes callous, peevish and hard. The wife bristles in self-defence. They develop an unnatural growth and sharpness of teeth, and the house is haunted by ugliness and domestic brawls. This is not what the family circle should be. If rude to any, let it be to some one he does not love—not to wife, brother or parent. Let one of the loved ones be taken away, and memory recalls a thousand sayings to regret. Death quickens recollection painfully. The grave cannot hide the white faces of those who sleep. The coffin and green ground are cruel magnets. They draw us farther than we would go. They force us to remember. A man never sees so far into human life as when he looks over a wife's or mother's grave. His eyes get wondrous clear, then, and he sees as never before what it is to love and be loved ; what it is to injure the feelings of the loved. It is a pitiable picture of human weakness when those we love best are treated worst.

---

Rub weak spines with a mixture of salt, tablespoonful of brandy, and tea cup of water.

---

When the eyes are tired and weak, if they are bathed in slightly saline water they will soon become soothed.

---

Any ordinary sticking plaster is good for corns, as it keeps the surface soft, and prevents the shoe from rubbing.

---

To insure good refreshing sleep, eat a light supper, forget the cares of the day, retire at a regular hour and never sleep during the day.

---

Compressed Air as Motive Power in France.—The use of compressed air as a motive power for tramways in France is extending. The system adopted is that invented by M. Mekarski, director of the Nantes tramways, which have been open since 1879. Two years ago the system was successfully applied on the tramways at Nogent, in the neighborhood of Paris, and more recently on those of Berne and Limoges. This year it will be substituted for horse power on the tramways of Lyons. The inventor asserts that his system is far more economical than horse traction,—the cost of coal per day of a machine equal to 8 or 10 horse power being only $1.00,—much cheaper than electricity or steam power, and that machinery is simple and does not require a skilled mechanic to control it. The British consul at Nantes, in a recent report, states "that the tramways of that town, which are worked by the system of M. Mekarski, alluded to above, continue to give satisfaction. The cars are comfortable and run smoothly with little noise. They do not interfere with the general traffic in the streets, and their immunity from accidents is remarkable. The average speed is about eight miles per hour ; but it can be easily increased or moderated, and in case of need an almost instantaneous stoppage effected."

### TEN GOOD THINGS TO KNOW.

1. That milk which is turned or changed may be sweetened and rendered fit for use again by stirring in a little soda.

2. That salt will curdle new milk; hence, in preparing milk porridge, gravies, etc., the salt should not be added until the dish is prepared.

3. That fresh meat, after beginning to sour, will sweeten if placed out of doors in the cool of night.

4. That clear boiling water will remove tea stains and many fruit stains. Pour the water through the stain, and thus prevent it spreading over the fabric.

5. That ripe tomatoes will remove ink and other stains, from white cloth; also from the hands.

6. That a tablespoonful of turpentine boiled with white clothes will aid in the whitening process.

7. That boiled starch is much improved by the addition of a little sperm salt, or gum arabic dissolved.

8. That beeswax and salt will make rusty flat-irons as clean and smooth as glass. Tie a lump of wax in a rag and keep it for that purpose. When the irons are hot, rub them first with the wax rag, then scour with a paper or cloth sprinkled with salt.

9. That blue ointment and kerosene, mixed in equal proportions and applied to the bedsteads, is an unfailing bedbug remedy, as a coat of whitewash is for the walls of a log house.

10. That kerosene will soften boots or shoes that have been hardened by water, and render them as pliable as new.

---

Sometimes a patent is worth something. Five years ago a poor inventor of Rochester, N. Y., was hawking about among lamp makers a new patent burner, without success. He was pooh-poohed and discouraged. Finally, a Mr. C. S. Upton, who had a good nose for a trade, took it in hand, and now the royalties paid on the lamps made under the patent, aggregate a fortune yearly. Over a million of "Rochester" lamps have been sold, and the proprietor has in New York the largest lamp store in the world, with branches in Paris and Chicago.

### A WISH ON THE MOON.

By M. D. Frazar.

Up in the evening sky
There gleams a faint new moon;
It floats so far and high,
This silver thread of June!

Into my thought there springs
A simple childhood's creed,
That earnest wishing brings
On each new moon its meed.

Protect my love, I pray,
Send happy days and light,
Nor any dim and gray,
And only moon-lit night.

Shed o'er her tender beams
Of rapture, true and deep,
Till all her life but seems
A harvest rich to reap.

